# Self-perceived general health and its impact on oral health in the U.S. adult population: NHANES 2015–2018

**DOI:** 10.3389/froh.2025.1590604

**Published:** 2025-07-14

**Authors:** Fahad AlAli, Abdulrahman Al-Safi, Al-Jawharah Al-Ajmi, Fatemah Alshammari, Sarah Saqer, Woroud Al-Sulimmani, Ahmed Balkhoyor, Hend Alqaderi, Hesham Alhazmi

**Affiliations:** ^1^ West Mubarak AlKabeer Polyclinic, Ministry of Health, Mubarak Al-Kabeer, Kuwait; ^2^ Hawalli West Poly Clinic, Hawalli, Kuwait; ^3^ Meteb Al-Shalahi Polyclinic, Ministry of Health, Al-Farwaniyah, Kuwait; ^4^ West Mishref Polyclinic, Ministry of Health, Hawalli, Kuwait; ^5^ Department of Oral and Maxillofacial Surgery, Jahra Hospital, Ministry of Health, Al-Jahra, Kuwait; ^6^ Department of Preventive Dentistry, Faculty of Dentistry, Umm Al-Qura University, Makkah, Saudi Arabia; ^7^ Department of Public Health and Community Service, Tufts University School of Dental Medicine, Boston, MA, United States; ^8^ Dasman Diabetes Institute, Sharq, Kuwait

**Keywords:** overall wellbeing, oral health, DMFT, dental decay, general health

## Abstract

**Introduction:**

Oral health is crucial to overall well-being and is often described as a “window to general health” due to the strong bidirectional relationship between the two. This paper aims to assess the relationship between self-perceived general health and oral health among U.S. adults.

**Methods:**

This study analyzed data from the 2015–2018 NHANES, comprising 11,566 U.S. adults. Statistical analyses included weighted percentages, chi-square tests, and logistic regression to evaluate the relationships between self-perceived general health status and oral health predictors.

**Results:**

Each unit increase in DMFT (decayed, missing due to caries, and filled teeth) resulted in a 2% increase in the odds of reporting fair to poor health compared with excellent to good health (*p* < 0.01). Additionally, for each additional decayed permanent tooth and each missing tooth, the odds of reporting fair to poor health compared with excellent to good health increased by approximately 38% (*p* = 0.004) and 43% (*p* = 0.010), respectively.

**Conclusion:**

This study suggests that higher DMFT scores, untreated dental decay, and missing teeth are associated with poorer self-perceived general health among U.S. adults. We recommend incorporating oral health assessments into general health check-ups, raising public awareness about their connection, and improving collaboration between medical and dental professionals to enhance patient care and preventive measures.

## Introduction

In recent years, dentists have increasingly focused on enhancing the overall health of their patients by prioritizing oral health (1, 2). The oral cavity is often referred to as the “window to general health” because of the well-established, bidirectional relationship between oral health and overall well-being (3, 4). Considering the advancements in preventive and therapeutic modalities within dentistry, oral diseases remain a substantial burden for socioeconomically disadvantaged communities, thus reinforcing long-standing disparities in oral health (5). A general surgeon report provided a comprehensive in-depth exploration of oral health, emphasizing its vital role in maintaining and promoting overall health and well-being (6). While a causal relationship between oral health and general health is still unconfirmed, comorbidities due to common risk factors appear to be a more acceptable explanation in view of the current evidence, which highlights the importance of integrating oral health, general health policies, and health-promoting interventions (7, 8). In addition, the findings highlight the importance of considering oral health status among individuals with compromised medical conditions (7, 9, 10). A substantial body of research demonstrates that the oral cavity plays a vital role, as it is essential for overall health, considering the complex relationship between oral diseases and various systemic conditions (11, 12). Concurrently, numerous public health scholars have advocated for dental practitioners to consider the contextual environment of patients more thoroughly, as well as to address the social determinants that influence their health, disease prevalence, and accessibility to healthcare services (13). Indeed, studies have shown that mental health and oral health may be correlated, with associations demonstrated between mental health problems and tooth loss, periodontal disease, and tooth decay (14). Population studies consistently support associations between poor oral (periodontal) health and systemic diseases such as cardiovascular disease (CVD) (15) and diabetes (16). Furthermore, cumulative evidence suggests an association between oral health and pulmonary disease, specifically chronic obstructive pulmonary disease (COPD) and pneumonia (17). Current evidence, of mixed quality, suggests several associations between oral diseases and diabetes mellitus (18–20).

A study utilizing data from the National Health and Nutrition Examination Survey (NHANES) conducted from 2017-March 2020 revealed that approximately 21% of dentate adults aged 20–64 years had one or more carious permanent teeth. Among adults aged 20–64 years with one or more decayed, missing, or filled teeth (DMFT >0), the average counts were as follows: 0.7 decayed teeth (DT), 6.0 filled teeth (FT), and 2.0 missing teeth due to disease (MT). These findings highlight significant public health concerns related to dental health disparities in the U.S. adult population (21). Several studies have examined the connection between oral health and various systemic diseases. One study by Shah et al., which analyzed NHANES data from 2009 to 2014, showed that individuals with current asthma were more likely to experience dental caries and have untreated dental caries than those without a history of asthma (22). Another study by Aldosari et al. used NHANES data from 2015 to 2018 to explore the relationship between diabetes mellitus and dental outcomes, including caries and missing teeth (23). Their findings indicated that adults with diabetes had a greater likelihood of developing both coronal and root caries than nondiabetic participants did (23). Additionally, adults with diabetes have a greater average number of missing teeth than their nondiabetic counterparts do (23). Additionally, a study investigating the association between self-reported depressive symptoms and oral diseases in U.S. adults utilizing NHANES data from 2009 to 2014 revealed that more than one-third of adults with moderate depressive symptoms had teeth with untreated dental caries (24).

The mouth is a gate into the body, such that systemic conditions and side effects of medical treatments may first manifest orally. However, the lack of integration between the dental-oral region and the broader body has hindered the timely recognition of these signs and symptoms (12). While several studies have examined the link between oral health and systemic diseases (17–20), our study aims to assess the relationship between self-perceived general health and dental caries experience (DMFT), as well as the number of untreated dental caries and missing teeth, using NHANES data to evaluate this at a national level.

## Materials and methods

Our study utilized data from the 2015–2018 NHANES, a cross-sectional study conducted by the U.S. Centers for Disease Control and Prevention. NHANES utilizes a multistage probability sampling method to obtain a representative sample of the U.S. population. The process begins by identifying Primary Sampling Units (PSU), which are typically counties, stratified by factors such as urban or rural status and geographic region. Within each PSU, smaller units (e.g., blocks) are selected, followed by a random selection of households and individuals within those households. The dataset included 11,566 adult participants from the United States. Data collection involved clinical examinations and demographic information obtained through standardized questionnaires. The dental examination conducted in NHANES occurs in a mobile examination center and is performed by licensed dentists. This oral health assessment utilizes visual-tactile method and does not involve x-rays. During the evaluation, all teeth are counted and assessed for dental caries.

### Covariate

Five variables were analyzed in this study: sex, race/ethnicity, family income categories based on Federal Poverty Levels (FPLs), age, education level, and current smoking status. Gender was categorized as male or female. Age was stratified into three groups: 19–44 years, 45–59 years, and 60 years and above. Race/ethnicity was classified into five categories: Mexican American and other Hispanic, Non-Hispanic White, Non-Hispanic Black, Non-Hispanic Asian, and Other. Education level was grouped into three categories: less than high school, high school, and more than high school education. Family income was divided into four categories: <100%, 100%–199%, 200%–399%, and ≥400%. Smoking status was divided into current smoker or not current smoker.

### Outcome variable

The outcome measured was participants’ self-reported health status, assessed through the question: “How would you rate your overall health?” Respondents could choose from options ranging from “excellent” to “poor”. This variable was categorized as a binary outcome, divided into two groups: “excellent to good” and “fair to poor”.

### Oral health predictors

− DMFT index: Used to measure the caries experience.
− untreated dental caried and number of missing teeth: Indicators of the burden of dental disease

### Statistical analysis

Complex survey design was utilized for the NHANES data, and the results were weighted to account for clustering and stratification. Additionally, we conducted analyses on combined data from two NHANES cycles and adjusted the weights by dividing them by the number of cycles. Descriptive statistics included the count and weighted percentage of the total population by demographic variables. The weighted percentages were reported for each demographic variable according to self-perceived general health status. The chi-square test was employed to assess relationships among variables. Logistic regression analyses were conducted to evaluate the associations between self-perceived general health status and the main predictors: DMFT score, untreated dental caries, and the number of missing teeth. Adjusted odds ratios (ORs) and 95% confidence intervals (CIs) are reported. A *p* value of less than 0.05 was considered statistically significant. Stata 18 was used to analyze the data.

## Results

Table 1 shows the demographic variables related to self-perceived general health among adults in the U.S. from 2015 to 2018. Most of the respondents were females (51.83%), aged 19–44 years (44.94%), and identified as non-Hispanic White (62.81%). With respect to education, the majority (63.12%) had completed education beyond high school. In terms of poverty income, 37.26% were at 400% and above the FPL. With respect to current smoking status, 58.08% were nonsmokers. Among the total population, 81.48% reported excellent to good general health, whereas 18.52% reported fair to poor general health. Among males, 8.71% reported fair to poor general health, whereas 9.81% reported poor general health for females. In terms of age, 6.9% of those aged 19–44 reported fair to poor general health. Additionally, 9.18% of non-Hispanic White individuals reported fair to poor general health. With respect to educational level, 7.82% of individuals with more than a high school education report fair to poor general health. In terms of family income, 5.47% of the participants between 100% and 199% of the FPL reported fair to poor general health. Finally, 12.55% of the current smokers reported fair to poor general health. The chi-square test results indicate significant associations (*p* < 0.05) between self-perceived general health outcomes and age, race/ethnicity, educational level, family income, and current smoking status.

**Table 1:** Descriptive analysis of self-perceived general health by demographic variables among U.S. adults from 2015 to 2018.

Variable	Total (n)	Total (%)^a^	Excellent to good (%)^a^	Fair to poor (%)^a^	*P*-value^b^
Gender	11,566		81.48%	18.52%	
Male	5,592	48.17%	39.46%	8.71%	0.316
Female	5,974	51.83%	42.02%	9.81%	
Age (years)					
19–44	4,800	44.94%	38.04%	6.9%	<0.01
45–59	2,715	26.89%	21.49%	5.4%	
60 and above	4,051	28.17%	21.96%	6.21%	
Race/Ethnicity					
Mexican American and other Hispanic	3,117	15.73%	10.82%	4.91%	<0.01
Non-Hispanic White	3,871	62.81%	53.63%	9.18%	
Non-Hispanic Black	2,547	11.42%	8.91%	2.51%	
Non-Hispanic Asian	1,532	5.84%	5.02%	.83%	
Others	499	4.19%	3.1%	1.09%	
Educational level					
Less than high school	2,481	12.93%	7.72%	5.21%	<0.01
High school	2,561	23.95%	18.46%	5.5%	
Some college and above	6,228	63.12%	55.3%	7.82%	
Family income categories based on FPL^c^					
<100%FPL	2,118	13.79%	8.95%	4.84%	<0.01
100%–199% FPL	2,796	20.64%	15.17%	5.47%	
200%–399% FPL	2,719	28.31%	23.85%	4.47%	
400% + FPL	2,465	37.26%	34.05%	3.21%	
Current smoker					
No	2,660	58.08%	46.87%	11.2%	<0.01
Yes	2,121	41.92%	29.38%	12.55%	

^a^ Weighted percentages.

^b^ Chi square test.

^c^ Federal Poverty Levels.

Table 2 presents the results of a logistic regression model assessing the link between DMFT and self-perceived general health among the U.S. adults. The table indicates that for each unit increase in DMFT, the odds of reporting fair to poor general health compared with excellent to good general health increased by 2% (*p* < 0.01, 95% CI: 1.01–1.03). Table 3 presents the results of a logistic regression model assessing the link between untreated dental decay and self-perceived general health among the U.S. adults. The table indicates that for each unit increase in untreated dental decay, the odds of reporting fair to poor general health increased by approximately 38% compared with excellent to good general health (*p* = 0.004, 95% CI: 1.12–1.70). Table 4 presents the results of a logistic regression model assessing the link between missing teeth and self-perceived general health among the U.S. adults. The table indicates that for each additional missing tooth, the odds of reporting fair to poor general health increased by 43% compared with excellent to good general health (*p* = 0.010, 95% CI: 1.10–1.86). The analyses were adjusted for age, sex, race/ethnicity, educational level, family income, and current smoking status.

**Table 2:** Association between DMFT and self-perceived general health among U.S. adults from 2015 to 2018.

Variable	Odds ratio	*P*-value^b^	95% Cl^c^
DMFT^a^	1.02	<0.01	(1.01–1.03)
Age (years)			
19–44	reference	reference	reference
45–59	1.62	<0.01	(1.25–2.10)
60 and above	1.72	<0.01	(1.15–2.56)
Gender			
Male	reference	reference	reference
Female	1.05	0.58	(0.88–1.25)
Race/Ethnicity			
Mexican American and other Hispanic	reference	reference	reference
Non-Hispanic White	1.70	<0.01	(1.32–2.17)
Non-Hispanic Black	1.19	0.216	(0.90–1.59)
Non-Hispanic Asian	1.01	0.969	(0.66–1.53)
Others	1.56	0.137	(0.86–2.83)
Educational level			
Less than high school	reference	reference	reference
High school	0.64	<0.01	(0.47–0.86)
Some college and above	0.46	<0.01	(0.37–0.59)
Family income categories based on FPL^d^			
<100% FPL	reference	reference	reference
100%–199% FPL	0.72	<0.01	(0.57–0.90)
200%–399% FPL	0.50	<0.01	(0.34–0.72)
400% + FPL	0.23	<0.01	(0.15–0.33)
Current smoker			
No	reference	reference	reference
Yes	1.55	<0.01	(1.20–2.01)

^a^ Decayed, missing due to caries, and filled teeth.

^b^ Logistic regression analysis.

^c^ Confidence interval.

^d^ Federal poverty levels.

**Table 3:** Association between untreated dental caries and self-perceived general health among U.S. Adults from 2015 to 2018.

Variable	Odds ratio	*P*-value^a^	95% Cl^b^
Untreated dental caries	1.38	0.004	(1.12–1.70)
Age (years)			
19–44	reference	reference	reference
45–59	1.75	<0.01	(1.33–2.31)
60 and above	2.09	<0.01	(1.42–3.07)
Gender			
Male	reference	reference	reference
Female	1.10	0.264	(0.93–1.29)
Race/Ethnicity			
Mexican American and other Hispanic	reference	reference	reference
Non-Hispanic White	1.66	<0.01	(1.29–2.14)
Non-Hispanic Black	1.12	0.432	(0.84–1.51)
Non-Hispanic Asian	0.99	0.969	(0.66–1.49)
Others	1.55	0.143	(0.85–2.81)
Educational level			
Less than high school	reference	reference	Reference
High school	0.64	<0.01	(0.47–0.87)
Some college and above	0.48	<0.01	(0.37–0.61)
Family income categories based on FPL^c^			
<100% FPL	reference	reference	Reference
100%–199% FPL	0.74	.013	(0.58–0.93)
200%–399% FPL	0.52	<0.01	(0.35–0.76)
400% + FPL	0.24	<0.01	(0.17–0.35)
Current smoker			
No	reference	reference	reference
Yes	1.52	<0.01	(1.181419–1.97)

^a^ Logistic regression analysis.

^b^ Confidence interval.

^c^ Federal Poverty Levels.

**Table 4:** Association between missing teeth and self-perceived general health among U.S. adults from 2015 to 2018.

Variable	Odds ratio	*P*-value^a^	95% Cl^b^
Missing teeth	1.43	0.010	(1.10–1.86)
Age (years)			
19–44	reference	reference	reference
45–59	1.57	<0.01	(1.21–2.05)
60 and above	1.78	<0.01	(1.22–2.57)
Gender			
Male	reference	reference	reference
Female	1.06	0.542	(0.88–1.26)
Race/Ethnicity			
Mexican American and other Hispanic	reference	reference	reference
Non-Hispanic White	1.62	<0.01	(1.25–2.10)
Non-Hispanic Black	1.15	0.343	(0.86–1.53)
Non-Hispanic Asian	0.95	0.814	(0.64–1.43)
Others	1.55	0.128	(0.87–2.76)
Educational level			
Less than high school	reference	reference	reference
High school	0.66	<0.01	(0.48–0.89)
Some college and above	0.48	<0.01	(0.38–0.62)
Family income categories based on FPL^c^			
<100% FPL	reference	reference	reference
100%–199% FPL	0.74	0.012	(0.58–0.93)
200%–399% FPL	0.51	<0.01	(0.35–0.74)
400% + FPL	0.24	<0.01	(0.16–0.34)
Current smoker			
No	reference	reference	reference
Yes	1.56	<0.01	(1.21–2.02)

^a^ Logistic regression analysis.

^b^ Confidence interval.

^c^ Federal poverty levels.

## Discussion

Our study assessed the associations between self-perceived general health and oral health among U.S. adults using NHANES data. The findings suggests that adults with higher DMFT scores, untreated dental decay, and missing teeth are associated with poorer self-perceived general health. Indeed, given the substantial advancements achieved in preventive and therapeutic methodologies within the domain of dentistry, our data shows that oral diseases continue to represent a significant obstacle and affects the general heath.

These findings align with the literature, confirming the relationship between higher DMFT scores, missing teeth, and poorer overall health, as seen in studies by Key et al. (25) and Sadiq et al. (26) They reported that adults with untreated dental caries had a greater risk of mortality than did those with no dental caries. The study also supports the notion that untreated dental caries contributes to increased mortality risk and that systemic conditions such as diabetes negatively affect oral health, as highlighted by Mahdieh et al. (27) Additionally, Bahanan et al.'s findings (28) linked food insecurity with higher rates of untreated dental caries, as they identified a connection between food insecurity and higher rates of untreated dental caries. This is reflected in our study and observed in the literature, as lower income levels correlate with poorer health, highlighting the impact of socioeconomic factors (28–30).

A study aimed to investigate the relationships between general health and lifestyle factors and oral health outcomes, and a population analysis involving 37,330 patients in the UK revealed that major health problems were significantly associated with lower oral health scores (31). These findings suggest that general dental practitioners may play a crucial role in influencing patients' lifestyle and health as part of their efforts to maintain optimal oral health. Another study aimed to examine the impact of oral health on the overall health and well-being of the geriatric population over 65 years old in the United States. Using data from the National Health and Nutrition Examination Survey (NHANES) 2015–2016, the analysis revealed statistically significant relationships between oral health and various aspects of health, including physical and mental health, energy levels, and systemic diseases (32). This evidence underscores the importance of addressing the oral health needs of elderly individuals, particularly in the context of patient-centered and value-based care. A systematic review aimed at examining longitudinal studies on the association between oral health and frailty reported significant associations between frailty and various oral health indicators (33). These findings emphasize the importance of oral health as a predictor of frailty in older adults. As the mouth is a gateway into the body, our study recommended that dental practitioners take into account the context of their patients more deeply, as well as address the oral heath condition that impact their general health.

Finally, our study has several limitations, primarily the use of NHANES data collected at a single point in time, which makes it difficult to measure causal relationships and track changes over time. However, it also highlights key strengths, such as the analysis of national data that incorporate multiple demographic variables and the assessment of various oral health outcomes. This study underscores the importance of maintaining dental health for overall well-being, warranting the development of public health campaigns to increase awareness of the link between oral and general health. Increased access to preventive dental care, especially in underserved communities, is essential, as is the integration of dental care with primary healthcare services. Future research should focus on longitudinal studies to explore causal relationships, investigate diverse populations, evaluate specific dental health interventions, and examine the biological mechanisms connecting oral and systemic health.

## Conclusion

Our study suggests that higher DMFT scores, untreated dental decay, and missing teeth are associated with poorer self-perceived general health among U.S. adults. We recommend integrating oral health assessments into general health check-ups and increasing public awareness campaigns about the connection between oral health and overall health. Additionally, fostering collaboration between medical and dental professionals might enhance patient care and promote preventive dental measures.

## Data Availability

Publicly available datasets were analyzed in this study. This data can be found here: https://wwwn.cdc.gov/nchs/nhanes/continuousnhanes/default.aspx?BeginYear=2017.
